# Synergistic induction of apoptosis in a cell model of human leukemia K562 by nitroglycerine and valproic acid

**DOI:** 10.17179/excli2019-1581

**Published:** 2019-08-15

**Authors:** Shahin Aalaei, Mehdi Mohammadzadeh, Yaghub Pazhang

**Affiliations:** 1 Department of Biology, Faculty of Sciences, Urmia University, Urmia, Iran

**Keywords:** apoptosis, combination therapy, cytotoxicity, K562 cells, nitroglycerine, valproic acid

## Abstract

Nitroglycerin (NG), a nitric oxide donor, and valproic acid (VPA), an inhibitor of histone deacetylases, have impressive effects on numerous cancer cell lines. This study intended to evaluate synergistic effects of NG and VPA on cell viability and apoptosis in K562 cells. K562 cells were cultured in RPMI-1640 supplemented with 10 % heat-inactivated FBS. They were treated with different doses of NG, VPA and cisplatin for 24, 48, and 72 h, and MTT assay was performed to analyze cell viability. Also, Peripheral blood mononuclear cells (PBMC) were cultured in RPMI-1640 media and incubated with NG (200 μM), VAP (100 μM), NG+VPA (150 μM) and cisplatin (8 μM) to evaluate cytotoxicity. IC_50_ of the drugs, when they were applied separately and in combination, were calculated using the COMPUSYN software. DNA electrophoresis, TUNEL assay, and Hoechst staining were performed to investigate apoptosis induction. RT-PCR was used for the evaluation of apoptotic genes expression. The results of the MTT assay showed that cell viability decreased at all applied doses of NG and VPA. It was noticed that the cytotoxic effects of these drugs were dose- and time-dependent. Based on the COMPUSYN output, the combination of the drugs (VPA and NG) in a certain ratio concentration synergistically decreased cell viability. Cisplatin significantly decreased cell viability of PBMCs and K562 cells. Also, the combination drug had cytotoxic effect and significantly reduced viability of K562 cells compared with PBMCs and control cells. In the target cells treated with this combination, Bax and caspase-3 expression increased but Bcl-2 expression decreased. These results suggest that NG, VPA, and their combination decreased cell viability and induced apoptosis via the intrinsic apoptotic pathway. This study suggests that this combination therapy can be considered for further evaluation as an effective chemotherapeutic strategy for patients with chronic myeloid leukemia.

## Introduction

The main reason for failure in the treatment of cancer is the occurrence of resistance to chemotherapy. However, chemotherapy is still an effective approach for treating cancer. For the majority of anti-cancer drugs, apoptosis appears to be initiated by the extrinsic (death receptor) pathway or the intrinsic (mitochondrial) pathway. In the intrinsic apoptosis pathway, the Bcl-2 family of proteins acts as a central regulator. The Bcl-2 protein, as a member of the Bcl-2 family, resides in the outer mitochondrial membrane or on the endoplasmic reticulum. It removes pro-apoptotic members such as Bax from mitochondria, which ultimately prevents apoptotic cell death. Increased Bax expression in cancer cells induces cell death and eliminates tumor cells. In contrast, increased Bcl-2 expression prevents apoptosis and leads to tumor progression. It is generally believed that chemotherapeutic drugs eliminate cancer cells by inducing apoptosis (Letai, 2017[25]).

The discovery and synthesis of biologically active molecules with cytotoxic properties has been considered as a potential new therapy for destroying tumor cells. Targeting histone deacetylases (HDACs), which have important roles in regulating cell proliferation, cell migration, and cell death via their inhibitors is one of the goals in cancer drug development. A wide variety of HDAC inhibitors (HDACIs) have shown anticancer effects used either singly (Wetzel et al., 2005[45]) or in combination with other agents (Seah et al., 2018[34]; Almenara et al., 2002[2]). Due to the sensitivity of tumor cells and relative resistance of normal cells to HDACIs, inhibiting these proteins is highlighted not only in clinical treatments but also in chemoprevention of cancer (Dong et al., 2018[11]; Ahrens et al., 2016[1]). Therefore, we suggest that inhibiting these targets provides normal cells with compensatory capabilities and relative resistance to induced cell death compared to the sensitive cancer cells. VPA, which belongs to the class of aliphatic acids, has been recently shown to inhibit HDACs activity, have antiproliferative and anticancer effects, and to be responsible for tissue-dependent changes in the transcriptome (Matthews et al., 2001[27]; Ho et al., 2012[18]; Frederiksen et al., 2007[13]; Brodie and Brandes, 2014[5]; Van Leeuwen et al., 2011[43]; Heers et al., 2018[16]; Witt et al., 2013[46]; Yarmohamadi et al., 2018[51]; Ni et al., 2017[28]). In addition to its effect on histone proteins, VPA can also influence the protein levels of non-histone proteins such as p53 (Cook et al., 2004[8]), Ku70 (Jordan et al., 2000[22]), Bcl-2, Bax, and caspase-3 that are important in cancer growth, apoptosis, DNA repair, differentiation, and cell cycle control (Huang et al., 1999[19]; Takabuchi et al., 2004[38]; Thomas et al., 2004[41]). Various studies have shown that VPA in combination with other drugs has increased anticancer effects. The combinatory effects of VPA and Fluvastatin demonstrate that they act synergistically in inducing γ-H2AX and apoptosis accompanied by higher acetylated histones H3 and H4 in GBM8401 cells. In addition, it has been revealed that VPA in combination with Mitomycin C consistently induces synergistic growth inhibition and decreases viability of colon carcinoma cells (Friedmann et al., 2006[14]). Various studies show that the combination of VPA and Metformin (MET) can synergistically decrease the viability of prostate cancer cells more efficiently than either drug used individually. Moreover, MET combined with VPA reduced proliferation and induced apoptosis in tumor cells (Tran et al., 2017[42]). Several studies have shown that combination of Cytarabine and VPA has significant anticancer effects in non-acute myeloid leukemia cases. Moreover, Cytarabine and VPA cooperatively induced DNA double-strand breaks, reflected in induction of γ-H2AX and apoptosis, accompanied by activation of caspase-9 and caspase-3. In addition, VPA induced Bim expression and short hairpin RNA knockdown of Bim resulted in significantly decreased apoptosis induced by Cytarabine alone or Cytarabine plus VPA. These findings help to better understand the mechanism of action of HDACIs in combination with other drugs for treatment of cancers (Xie et al., 2010[48]). 

Nitroglycerin (NG) is a nitric oxide (NO) donor which can improve the response to chemotherapy, reverse resistance to chemical agents, and have pro-apoptotic and anti-efflux effects. NG belongs to a group of drugs called nitrates that exert their effect through being converted to nitric oxide (NO) in the body by mitochondrial aldehyde dehydrogenase. It is generally accepted that the main mechanism of action of NG is via NO production (Sukhatme et al., 2015[37]). NO is a ubiquitous signaling, regulatory, and effector molecule with diverse effects on the human body. In relation to cancer, NO has dichotomous pro-and anti-tumorigenic effects, depending on concentration, microenvironment, and cell type. In particular, low concentrations of NO (<100 nM) are associated with increases in angiogenesis, proliferation, and resistance to apoptosis, whereas high NO concentrations (>500 nM) are associated with increased cytotoxicity and apoptosis (Laggner et al., 2009[24]; Rogers and Holen, 2011[32]; Santini et al., 2010[33]). Therefore, NO donors can provide conditions to reverse resistance of drug(s) and alter the effects of drugs even at lower doses in human cancers (Riganti et al., 2005[31]; Huerta, 2015[21]; Hickok and Thomas, 2010[17]). The results of various studies have demonstrated that combination therapy with NG during chemotherapy might enhance the response rate or chemosensitivity to specific chemical agents such as vinorelbine, cisplatin, docetaxel, and carboplatin in patients with cancer (He et al., 2016[15]; Yasuda et al., 2006[52]; Da Costa et al., 2018[9]). In the present study, we investigated the synergistic effects of NG and VPA and explored their cytotoxic effects on inducing apoptosis in human leukemia K562 cells. It is necessary to develop effective chemotherapies that enhance cell death in cancer cells at lower doses and with fewer side effects.

## Material and Methods

### Cell culture, treatment and cell viability assay

The human leukemic K562 cell line was purchased from the Iranian Pasteur Institute. The cells were cultured in RPMI-1640 (GIBCO, Life Technologies, Carlsbad, CA., USA) medium supplemented with glutamine, 10 % (v/v) heat-inactivated fetal bovine serum (FBS) (GIBCO, Life Technologies, Carlsbad, CA, USA) and 100 U/ml Penicillin and Streptomycin (KeyGen Biotech. Co. Ltd), and incubated at 37 °C in a humidified atmosphere containing 5 % CO_2_. The cells were sub-cultured thrice a week. K562 cells were treated with different concentrations of Cisplatin, Valproic acid and Nitroglycerin (purchased from Sigma) for 24, 48 and 72 h (Chavez-Blanco et al., 2006[7]). As well, peripheral blood mononuclear cells (PBMC) were isolated from healthy human volunteer by density gradient centrifugation as per standard procedure (Sharma et al., 2007[35]). PBMC (10^5^ cells/well) were cultured in RPMI-1640 media and treated with concentration of NG (200 μM), VAP (100 μM), NG+VPA (150 μM) and cisplatin (8 μM) for 72 h.

About 10^5^ K562 cells per well in 96-well plate were cultured and treated with various concentrations of Cisplatin (2, 4, 6 and 8 µM), NG (50, 100, 150 and 200 µM) and VPA (25, 50, 75 and 100 µM) to evaluate cytotoxicity for 24, 48, and 72 h using MTT assay. In the MTT assay method, 10 μl of MTT solution was added to each well and incubated at 37 °C for 2 h. In order to dissolve formazan crystals, 200 μl of DMSO (purchased from Sigma) was added to the wells and the wells were shaken at room temperature for 20 min. Absorbance was read by a microplate reader (BIOTEK) at 570 nm and cell viability was calculated for three replicates. For positive control, the cells were treated with different concentrations of Cisplatin. Also, PBMCs were cultured and incubated with NG (200 μM), VAP (100 μM), NG+VPA (150 μM) and cisplatin (8 μM) to evaluate cytotoxicity for 72 h using MTT assay. The data were reported as mean ± SD (Xu and Zhang, 2005[49]). 

### Synergism analysis and apoptosis assay

Combination index (CI) is widely used to quantify drug synergism based on the multiple drug effect equation of Chou-Talalay. In our study, the CI values were determined for each concentration of NG, VPA, and their combination (VPA+ NG) in cell proliferation assays using CalcuSyn or CompuSyn (Biosoft, Cambridge, UK). According to this index, CI < 1, CI = 1, and CI > 1 represent synergism, additive effect, and antagonism, respectively (Zhang et al., 2016[53]). Based on MTT assay data from the cytotoxic effect of VPA and NG on K562 cells, their IC_50s_ were calculated by COMPUSYN. Next, various concentrations of the combination drug were prepared according to their IC_50_. Eventually, their synergism was calculated by COMPUSYN based on Chou-Talalay theory.

To investigate the apoptosis-inducing effect, about 10^6^ cells were cultured and treated with an IC_50_ concentration of NG and VPA and 150 μM concentration of the combination drug. The K562 cells were cultured and treated with the drugs and then centrifuged at 1000 g for 5 min. After centrifugation, the lysis buffer was added to the cell pellets and DNA was extracted using the phenol-chloroform-isoamyl alcohol procedure. The extracted DNA was analyzed in 1 % agarose gel electrophoresis at 80 mV for 1 hour in the presence of ethidium bromide. Finally, the gel was visualized under UV illumination in a gel documentation system (Mahdavi et al., 2011[26]). After that, the TUNEL assay was performed using the protocol of TUNEL assay kit provided by the manufacturers. Briefly, the slides were predigested with 20 mg/ml proteinase K for 20 min and then incubated in a phosphate buffered saline solution (PBS) containing 3 % H_2_O_2 _for 10 min to block the endogenous peroxidase activity. They were then incubated with the TUNEL reaction mixture, fluorescein-dUTP (in situ Cell Death Detection Kit, Fluorescein, Roche, Germany), for 60 min at 37 °C based on the manufacturer's instructions. The slides were then rinsed three times with phosphate buffered saline (PBS). After that, they were stained with Hoechst 33342 (Sigma-Aldrich) for the chromogenic reaction. As a control for method specificity, the TUNEL reaction mixture step was omitted in negative control slides and nucleotide mixture in reaction buffer was used instead (Darzynkiewicz et al., 2008[10]; Zhang and Kiechle, 1997[55]). 

### Gene expression analysis

RNA was extracted by TRIzol reagent from untreated and treated cells. Then, cDNA was synthesized according to the cDNA synthesis kit protocol (Cat. No. A101161, Parstous Biotechnology). Bax, Bcl-2, Caspase-3, and GADPH primers were designed by Gene Runner software with the following sequence.

BAX Forward primer: 5′-CCTTTTCTACTTTGCCAGCAAAC-3′; 

BAX Reverse primer: 5′-GAGGCCGTCCCAACCAC-3′; 

BCL2 Forward primer: 5′-ATGTGTGTGGAGAGCGTCAACC-3′; 

BCL2 Reverse primer: 5′-GCATCCCAGCCTCCGTTATC-3′; 

Caspase-3 Forward primer: 5′-TTAATAAAGGTATCCATGGAGAACACT-3′; 

Caspase-3 Reverse primer: 5′-TTAGTGATAAAAATAGAGTTCTTTTGTGAG-3′;

GAPDH Forward primer: 5′-CGTCTGCCCTATCAACTTTCG-3′; 

GAPDH Reverse primer: 5′-CGTTTCT CAGGCTCCCTCT-3′.

PCR reaction was performed with the following steps using Master Mix (Thermo Scientific Catalog number: K0171): pre-denaturation for 5 min at 95 °C followed by 35 cycles of 10 sec denaturation at 95 °C, annealing/extension for 15 sec at 59 °C, and final extension for 20 sec at 72 °C. Afterwards, cDNA concentration was determined at 260 nm. For electrophoresis, 50 ng cDNA was loaded on a 1 % agarose gel at 85 mV for 45 min. Finally, the gel photo was captured by gel-documentation. Densitometric analysis of the bands was done by using PCR Gel analyzing software (Version: 2.00, ATP, Tehran, Iran). The control was set at 100 % and the experimental samples were compared to the control (Zheng et al., 2018[56]).

The experiments were repeated in triplicate and the data was reported as mean ± SD. The statistical significance of results was calculated using t-test in Excel and represented as p-value < 0.05.

## Results

### Nitroglycerine, Valproic acid, Cisplatin and combination drug reduced K562 cell viability 

**Figure 1:**
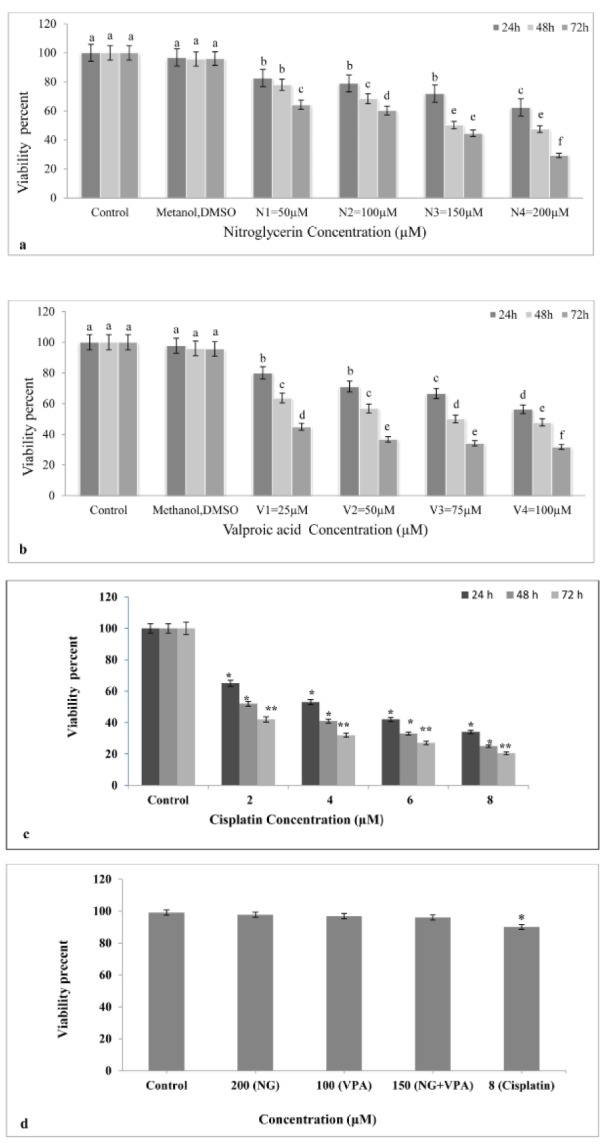
Figure1: Cytotoxic effect of Nitroglycerine and Valproic acid on K562 cell line. a: Effect of Nitroglycerine on K562 cell line. The cell viability has decreased after treatment with different concentration of nitroglycerine. The most cytotoxic effect was observed in 200 μM at 72 hours after treatment (p<0.05). b: Effect of Valproic acid on K562 cell line. Treatment with different concentrations of valproic acid decreased cell viability, as understood the drug effect is time- and dose-dependent. The most cytotoxic effect was observed in 100 μM at 72 hours after treatment. Different letters indicate significant differences between groups (p<0.05). c: Effect of cisplatin on K562 cell line as a positive control. Cisplatin in all applied concentrations reduce cell viability. d: Cytotoxic effects of NG (200 μΜ), VPA (100 μΜ), NG+VPA (150 μΜ) and Cisplatin (8 μM) on normal PBMC cells for 72 h (*)= p≤0.05, (**) = p≤0.01.

Results showed that viability of NG treated cells decreased in comparison with untreated cells. Moreover, NG influenced cell viability in a dose- and time-dependent manner 24, 48, and 72 h after treatment (Figure 1a(Fig. 1)). The maximum antiproliferative effect (70.7 %) was obtained after 72 h exposure to 200 µM NG. Furthermore, the IC_50_ of 158 µM was calculated for NG by CompuSyn.

According to the results, viability in VPA treated K562 cells decreased compared to the untreated K562 cells. The cytotoxic effect of VPA also depended on drug dose and time ( Figure 1b(Fig. 1)). Moreover, the maximum cytotoxicity (68.3 %) was obtained after 72 h exposure to 100 µM VPA. The IC_50_ of VPA was 80 μM as determined by CompuSyn.

Results indicated that all studied concentrations of Cisplatin (as positive control) reduced cell viability. Moreover, Cisplatin affected cell viability in a dose- and time-dependent manner 24, 48, and 72 h after treatment (Figure 1c(Fig. 1)). As shown in Figure 1d(Fig. 1), only the cisplatin (8μM) significantly decreased cell viability of PBMCs obtained from human blood (p<0.05).

**Figure 2:**
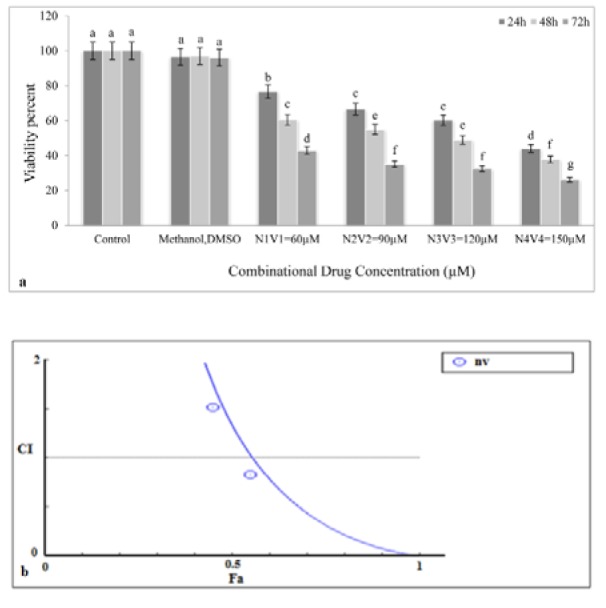
Synergism effect of combination drug (NG+VPA). a: Effect of combination drug on K562 cell viability. In all administered concentrations of combination drug the cell viability decreases and the most reduction in cell viability has been occurred in 150 μM at 72 hours. b: Synergism curve of combination drug. A combination index (CI) is estimated from dose-effect data of single and combined drug treatments. According to the combination index (CI), CI <1, CI = 1, and CI > 1 represent synergism, additive effect, and antagonism, respectively. Fa is fraction of cells that affected by treatment. Except for concentration with 150 µΜ (CI<1) in other used doses antagonism has been occurred (CI>1). Different letters indicate significant differences between groups (p<0.05).

To investigate synergistic effects of NG and VPA, various concentrations of the combined drug (NG+VPA) (60, 90, 120, and 150 µM) were prepared and added to the K562 cells in the culture medium. As shown in Figure 2a(Fig. 2), all concentrations significantly decreased growth rate of K562 cancer cells (p<0.05). The maximum cytotoxic effect was observed after 72 h exposure to the 150 µM concentration of the combination drug.

### Synergistic effects of NG and VPA on the viability and apoptosis of K562 cell line 

Based on the diagram obtained from CompuSyn (Figure 2b(Fig. 2)), NG and VPA showed synergistic effects only at their highest concentrations (150 µM) because the CI (combination index) value at this concentration was less than 1 but was higher than 1 at other concentrations (60, 90, and 120 µM), which suggested antagonism. The IC_50_ values showed that the potential anticancer property of NG was higher than that of VPA.

### Apoptosis induction after treatment with Nitroglycerine, Valproic acid and combination drug

**Figure 3:**
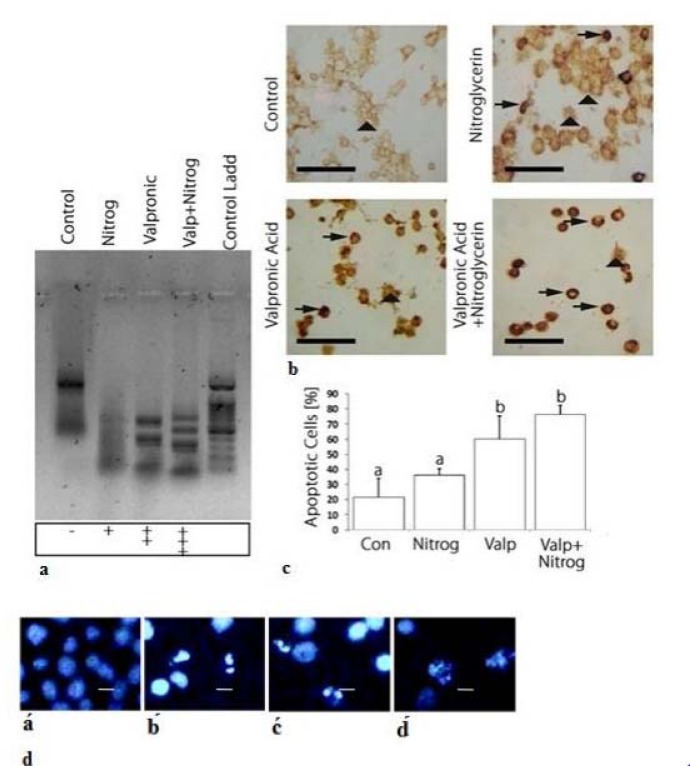
Apoptosis induction by nitroglycerine and valproic acid and their combination drug (VPA+NG). a: Quick detection of apoptotic DNA ladder and DNA fragmentation analysis by DNA electrophoresis in K562 cell line: Lane of control: untreated cells, Lane of Nitrog: treated cells with NG, Lane of Valproic: treated cells with VPA, Lane of Valp+Nitrog: treated cells with NG+VPA, Lane of Control Ladd: treated cells with standard molecular size marker. In treated cells, DNA fragemntation is occurred which is a key feature of apoptosis process. In valproic acid and combination drug (NG+VPA) lane DNA laddering occurs clearly, while in the nitroglycerine lane DNA laddering is low which indicates low apoptosis induction has been occurred. b: Tunnel staining of control and treated cells. In tunnel assay the dense staining is indicator of apoptotic cell. In control cells dense points is not observed, as a while in the treated cells the points are present and the most number of the dense points observed on combination drug (NG and VPA) treatment which indicates the most apoptosis induction in combination drug treatment. The vertical arrows show normal cells and the horizontal arrows represent apoptotic cells. c: Charts of apoptotic cells percentage in control and treated cells. Basis of tunnel assay results, the charts have been designed. d: K562 cells stained with Hoechst dye (33342) under fluorescence microscope. á: untreated cells, b́: treated cells with the combination drug (NG+VPA), ć: treated cells with VPA, d́: treated cells with NG. The Hoechst dye stains the nucleus by binding to DNA, in control cells the nucleus is not dense or fragmented, whereas in apoptotic cells it is dense and fragmented. In treated cells dense nucleus is observed which is an indicator of apoptosis process. Different letters indicate significant differences between groups. The scale bar represents 20 μm in Figure 3d (p<0.05).

Agarose gel electrophoresis of DNA extracted from K562 cells treated with NG, VPA, or the combined drug for 72 h are shown in Figure 3a(Fig. 3). As shown in the gel image, DNA fragmentation revealed that the drugs were able to induce apoptosis both individually and in combination. Our results demonstrated that drug combination caused greater DNA fragmentation in comparison with individual drug use.

The TUNEL assay revealed strong- and weak-staining intensity for apoptotic and non-apoptotic cells, respectively. As Figures 3b and 3c(Fig. 3) show, staining intensity was weak in the control cells, whereas strong staining was observed in the treated cells. More apoptotic cells were detected in the combination drug treatment compared to those in which NG or VPA was applied. Results indicated that the combination drug treatment was able to induce apoptosis more effectively than the individual use of the drugs.

Hoechst 33342 staining was used to detect apoptotic K562 cells by making observations through fluorescence microscopy. As illustrated in Figure 3d(Fig. 3), the morphological changes in drug-treated cells confirmed the induction of cell apoptosis. Microscopic observation of fragmented nuclei revealed that NG and VPA were capable of inducing apoptosis when used singly or in combination.

### Bax/Bcl-2 ratio increased after treatment with Nitroglycerine, Valproic acid and combination drug

**Figure 4:**
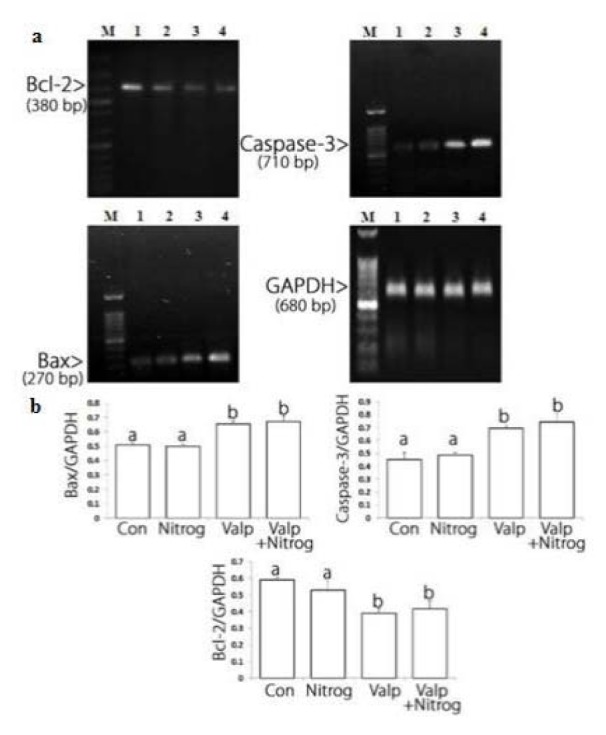
Bcl-2, Bax and caspase-3 mRNA level alterations in control and treated cells. a: Bcl-2 mRNA level decreases after treatment and the most reduction was observed in combination drug (VPA+NG) treatment. Bax and caspase-3 mRNA levels increase after treatment. Lane M: DNA marker, Lane 1: untreated cells, Lane 2: cells treated with Nitroglycerine (NG). Lane 3: cells treated with Valproic acid (VPA). Lane 4: cells treated with combination drug (NG+VPA). b: Charts of Bcl-2, Bax and caspase-3 mRNA alterations after treatment. Basis of electrophoresis results the chare have been designed. The most reduction in Bcl-2 mRNA level occurs in combination drug treatment, and the most elevation in Bax mRNA level occurs in combination drug treatment, the most caspase-3 mRNA level elevation was observed on valproic acid treatment. Different letters indicate significant differences between groups (p<0.05).

After the cells were treated with NG, VPA, and their combination, Bax mRNA levels rose at specific concentrations of the drug, especially at the highest concentration of the combination drug. The largest reduction in Bcl-2 mRNA level was observed in VAP treated cells. Moreover, the highest increase in Bax mRNA level was recorded in the samples treated with VPA. Altogether, Figure 4(Fig. 4) suggests that NG, VPA, and their combination were able to change the apoptotic genes expression. Based on the significant apoptosis induction of the combination drug compared to monotherapy and the VPA effects on Bcl-2 downregulation and caspase-3 upregulation, combination therapy may exert its main effect via altering caspase-3 and Bcl-2 protein levels.

## Discussion

The mitochondrial apoptotic pathway is regulated by pro-and anti-apoptotic members of the Bcl-2 protein family at the level of the mitochondria. The Bcl-2 family of proteins plays a key role in balancing the decision between cell survival and apoptosis, and escape of apoptosis is a hallmark of cancer. The pro-apoptotic members of the Bcl-2 family, such as Bim, Bid, Bad, PUMA, Noxa, can transmit the apoptotic stimuli by activating Bax and Bak. The anti-apoptotic members such as Bcl-2 and Bcl-xL counteract this process by binding to and neutralizing the pro-apoptotic proteins. After being activated, Bax and Bak form an oligomeric pore on the mitochondrial outer membrane (MOM), which is followed by the release of cytochrome c*. *Then they participate in the formation of the apoptosome with Apaf1 and caspase-9 and activation of caspase-3 (Prenek et al., 2017[29]). Results of previous studies showed that the resistance of K562 cells to etoposide-induced apoptosis was partially caused by the failure of Bax translocation to mitochondria. In this study, we found that K562 cells were sensitive to the combination of VAP and NTG (Figure 3(Fig. 3)). Apparently, in apoptotic cells, Bax upregulation, Bcl-2 gene downregulation, and cytochrome C release from mitochondria activate caspases-3 (Letai, 2017[25]; Rastogi et al., 2009[30]). Considering the downregulation of anti-apoptotic Bcl-2 protein, it can be inferred that mitochondria are directly involved in the regulation of K562 cell apoptosis. Under these conditions, mitochondrial proteins are released into the cell cytosol following the increase in permeability of the mitochondria membranes due to the formation of a channel by Bax protein. We showed that apoptosis induction in K562 cells took place via the intrinsic or mitochondrial apoptotic pathway (Figure 4(Fig. 4)). Moreover, the present study indicated that the combination drug was effective in synergistically reducing cell viability and inducing apoptosis (Da Costa et al., 2018[9]). Based on the results of this assessment, the combination of VPA and HDACI performed better than NG in inducing apoptosis. Therefore, HDAC inhibition could be a strategy for cancer treatment. Moreover, HDAC inhibitors showed anticancer effects in some cancer cell lines (Eckschlager et al., 2017[12]). This inhibitor decreases epithelial cell proliferation via downregulation p38 MAPK signaling pathway (Anand et al., 2018[3]). In other studies, it was observed that VPA could decrease proliferation of Metformin-resistant human renal cell carcinoma cells and inhibit invasiveness in bladder cancer (Wei et al., 2018[44]; Yagi et al., 2010[50]; Tang et al., 2004[39]). As shown in Figure 1b(Fig. 1), VPA significantly decreases cell viability compared to K562 cells not treated with it. Since the cytotoxic effect of this acid is dose- and time-dependent, it can reduce cell viability. TUNEL assay and Hoechst staining suggest that VPA induced apoptosis 72 h after the treatment. In the present study, VPA decreased cell growth by apoptosis induction in the K562 chronic myeloid leukemia (CML) cell line 72 h after treatment (Figure 1b(Fig. 1)). For positive control, the K562 cells were treated with various concentrations of Cisplatin that reduced their cell viability and growth at all the studied concentrations (Figure 1c(Fig. 1)) (Shruthi and Bhasker Shenoy, 2018[36]). Cytotoxic effects of NG (200 μΜ), VPA (100 μΜ), NG+VPA (150 μΜ) and Cisplatin (8 μM) was performed on normal PBMC and K562 cells for 72 h and it appeared that viability of combination drug treated cells significantly decreased in comparison with PBMCs and untreated cells (Figures 1a, b, c, d(Fig. 1) and 2a(Fig. 2)). As can be seen in Figure 4(Fig. 4), VPA induced apoptosis through decreasing Bcl-2 mRNA level and increasing either Bax or caspase-3 mRNA level.

NO is a mineral substance that plays physiological roles such as vasodilatation. It is produced in cells by nitric oxide synthase (NOS). NO reacts with reactive oxygen species and produces reactive nitrogen species that have cytotoxic effects and cause cell death. Considering their cytotoxic effects, NO donor agents would be a good candidate for cancer therapy (Huang et al., 2017[20]). As shown in Figure 1a(Fig. 1), NG reduced cell viability in a dose- and time-dependent manner so that its highest cytotoxic effect was observed 72 h after the treatment. Because of the cytotoxic effects of NO donors on cancer cells, they could induce apoptosis in cancer cell lines (Zhou et al., 2016[57]). Endogenous NO induced apoptosis in human melanoma cells (Kim, 2012[23]). NG induced apoptosis by reducing Bcl-2 mRNA level and through increasing BAX and caspase-3 mRNA levels (Figure 4(Fig. 4)) in the K562 cell line.

Combination therapy is a treatment in which two or more therapeutic agents are combined. Combination of two anticancer agents increases treatment efficacy compared to monotherapy because the combination drug targets key pathways in a characteristically synergistic or additive manner. This approach potentially reduces drug resistance and growth and induces apoptosis in cancer cells (Bayat Mokhtari et al., 2017[4]). VPA augments the cytotoxic effect of 5-Aza-2'-Deoxycytidine on human clear cell renal cell carcinoma (Xi et al., 2018[47]). It also synergistically induces apoptosis in Fluvastatin treated glioblastoma cells (Chang et al., 2017[6]). Furthermore, a combination of Metformin and VPA synergistically induced apoptosis and had an effective cytotoxic effect on prostate cancer (Zhang et al., 2015[54]). As can be seen in Figures 2a and 2b(Fig. 2), VPA synergistically reduced the viability of NG treated cells. Apoptosis induction in combination drug therapy was more effective compared to monotherapy (Figure 3(Fig. 3)). Therefore, the Bcl-2 mRNA level decreased and mRNA level of Bax and caspase-3 increased more in combination drug therapy than in monotherapy. As a result, VPA increased cytotoxic and apoptotic effects of NG in K562 cell line. NO released by NG may damage DNA. This will activate the p53 protein, decrease cell viability and induce apoptosis (Huerta, 2015[21]). In addition, VPA could induce p53 activation by its acetylation (Thakur et al., 2011[40]). The VPA and nitroglycerin synergistic effects might be due to their effect on p53 activation. These synergistic effects indicate that erythromyeloid leukemia may be treated more effectively by a combination of the two drugs compared to their individual use. However, further studies should be performed to investigate their combined use in cancer therapy. 

In conclusion, nitroglycerin, valproic acid, and their combination decrease cell viability and increase apoptosis in the K562 cell line. Nitroglycerin synergistically augments the cytotoxic effect of valproic acid on the K562 cell line and thereby effectively induces apoptosis via the intrinsic apoptotic pathway. The synergistic effect was induced by decreasing the Bcl-2 level and increasing the Bax and caspase-3 levels. This study suggests that this combination drug therapy is a good candidate for further evaluation as an effective chemotherapy for patients with chronic myeloid leukemia.

## Acknowledgement

We are grateful to the laboratory and technical services provided by the Faculty of Sciences, Urmia University, Urmia, Iran.

## Funding source

This study was supported by the Faculty of Sciences, Urmia University, Urmia, Iran.

## Disclosure statement

The authors declare that they have no conflict of interest.
